# Transcriptional repression of *frequency* by the IEC-1-INO80 complex is required for normal *Neurospora* circadian clock function

**DOI:** 10.1371/journal.pgen.1006732

**Published:** 2017-04-12

**Authors:** Kexin Gai, Xuemei Cao, Qing Dong, Zhaolan Ding, Yashang Wei, Yingchun Liu, Xiao Liu, Qun He

**Affiliations:** 1 State Key Laboratory of Agrobiotechnology and MOA Key Laboratory of Soil Microbiology, College of Biological Sciences, China Agricultural University, Beijing, China; 2 Department of Physiology, The University of Texas Southwestern Medical Center, Dallas, Texas, United States of America; Charité - Universitätsmedizin Berlin, GERMANY

## Abstract

Rhythmic activation and repression of the *frequency* (*frq*) gene are essential for normal function of the *Neurospora* circadian clock. WHITE COLLAR (WC) complex, the positive element of the *Neurospora* circadian system, is responsible for stimulation of *frq* transcription. We report that a C2H2 finger domain-containing protein IEC-1 and its associated chromatin remodeling complex INO80 play important roles in normal *Neurospora* circadian clock function. In *iec-1*^*KO*^ strains, circadian rhythms are abolished, and the *frq* transcript levels are increased compared to that of the wild-type strain. Similar results are observed in mutant strains of the INO80 subunits. Furthermore, ChIP data show that recruitment of the INO80 complex to the *frq* promoter is IEC-1-dependent. WC-mediated transcription of *frq* contributes to the rhythmic binding of the INO80 complex at the *frq* promoter. As demonstrated by ChIP analysis, the INO80 complex is required for the re-establishment of the dense chromatin environment at the *frq* promoter. In addition, WC-independent *frq* transcription is present in *ino80* mutants. Altogether, our data indicate that the INO80 complex suppresses *frq* transcription by re-assembling the suppressive mechanisms at the *frq* promoter after transcription of *frq*.

## Author summary

Circadian clocks organize inner physiology to anticipate changes in the external environment. These clocks are controlled by the oscillation of central clock proteins which form the central oscillator. Transcriptional regulation is a critical step in the regulation of the oscillation of these core proteins. In eukaryotes, chromatin remodeling is a common mechanism to regulate gene transcription by conquering or establishing nucleosomal barriers for the transcription machinery. Here, we showed that a C2H2 finger domain-containing protein IEC-1 and its associated chromatin remodeling complex INO80 are required for transcriptional repression of the core clock gene *frq* in the *Neurospora* circadian system. Moreover, the activator WHITE COLLAR (WC) complex is responsible for the transcriptional activation of *frq*; thus, considering the different timing of the transcriptional activation and suppression of *frq*, there must be a mechanism that coordinates the two opposite processes. We also demonstrated that the WC-mediated open state of the *frq* promoter facilitates the binding of INO80 to this region, which prepares for subsequent transcriptional suppression. Collectively, our data provide novel insights into the regulation of the *frq* gene and the circadian clock.

## Introduction

From the filamentous fungus *Neurospora crassa* to animals, circadian oscillation is a conserved mechanism based on an auto-regulatory feedback loop composed of negative and positive elements [1–6]. In *Neurospora*, the heterodimeric WHITE COLLAR (WC) complex, consisting of WC-1 and WC-2, acts as the positive element that binds to the *frq* promoter and activates *frq* transcription [7–13]. The negative elements FRQ and FRQ-interacting RNA helicase (FRH) form the FRQ/FRH complex and mediate the phosphorylation of WCs, which inhibits their WC complex activity and promotes the cytoplasmic localization of the WC complex [12,14–16]. Immediately following synthesis, FRQ is progressively phosphorylated by casein kinases (CKI and CKII) and other kinases throughout the subjective day and evening [12,17–20]. CKI participates in the regulation of both FRQ and the WCC [12]. The phosphorylation of FRQ by CKI increases the degradation rate of FRQ. FRQ protein also acts as a scaffold by bringing CKI to phosphorylate the WCC which leads to its inactivation and repression. Similar to CKI, many of the sites on FRQ protein can also be phosphorylated by CKII, which promotes its degradation [12,21]. While casein kinases regulate the stability of FRQ, the casein kinases are countered by multiple protein phosphatases, including PP1, PP2A and PP4 [22,23], The PP1 dephosphorylates and stabilizes FRQ protein while PP2a and PP4 activities influence *frq* transcription by dephosphorylating WC-2. Hyperphosphorylated FRQ is degraded through the ubiquitin-proteasome pathway [24]. However, a recent study showed that the cycle ends when FRQ is sufficiently hyperphosphorylated and becomes invisible to the circadian machinery [25]. When the activity of FRQ is not sufficient to suppress the activity of the WCs, *frq* transcription is reactivated by the WC complex. Recently, epigenetic modifications were reported to regulate *frq* transcription in *Neurospora*. SET-1 is required for normal expression of *frq* [26], and the SET-2 pathway is involved in the suppression of WC-independent *frq* transcription [27]. Moreover, antisense transcription was shown to inhibit sense expression by mediating chromatin modifications and premature termination of transcription in the *frq* locus [28].

Recent studies have shown that CLOCK:BMAL1 promote the removal of nucleosomes at its binding sites in mammalian clock genes during transcription activation [29]. Based on these results, nucleosomal barriers at the activator-binding sites should be established during transcriptional repression of the circadian cycle. A still unknown factor(s) may be responsible for the rhythmic incorporation of nucleosomes into chromatin at the activator-binding sites of the clock genes to establish rhythmic transcriptional repression.

Chromatin remodelers are capable of removing, destabilizing, ejecting, and restructuring nucleosomes by using the energy provided by ATP hydrolysis. In *Neurospora*, two ATP-dependent chromatin-remodeling factors, CLOCKSWITCH (CSW-1) and chromodomain helicase DNA-binding-1 (CHD1), have been reported to regulate *frq* transcription by modulating nucleosome density at the promoter region [7,30]. Recent studies have shown that SWI/SNF is recruited to the *frq* promoter by WC-1 to initiate *frq* transcription [31]. The INO80 complex is a highly conserved chromatin remodeler from yeast to humans [32]. INO80 can mobilize a mononucleosome to the center of a DNA fragment in vitro and remove histone variant H2A.Z from nucleosomes [33–36], which is involved in gene activation in yeast [37,38]. In yeast or mammalian systems, the recruitment of the INO80 complex by yeast Iec1 or mammalian Yin Yang 1 is a key event in gene regulation [39,40]. Moreover, the INO80 complex contributes to chromatin silencing of the boundaries of genes and heterochromatins [41]. However, little is known about the role of the INO80 complex in the suppression of protein-coding genes. Here, we demonstrate that the IEC-1-recruited INO80 complex is required for the *Neurospora* circadian clock system. WC-mediated transcriptional activation accounts for the rhythmic recruitment of the INO80 complex to the *frq* promoter; binding of the INO80 complex to the promoter region creates a dense chromatin environment and suppresses *frq* transcription. Loss of the INO80 complex leads to WC-independent *frq* transcription due to the accessible chromatin environment at the *frq* promoter.

## Results

### IEC-1 is required for normal circadian conidiation rhythm

**Fig 1:**
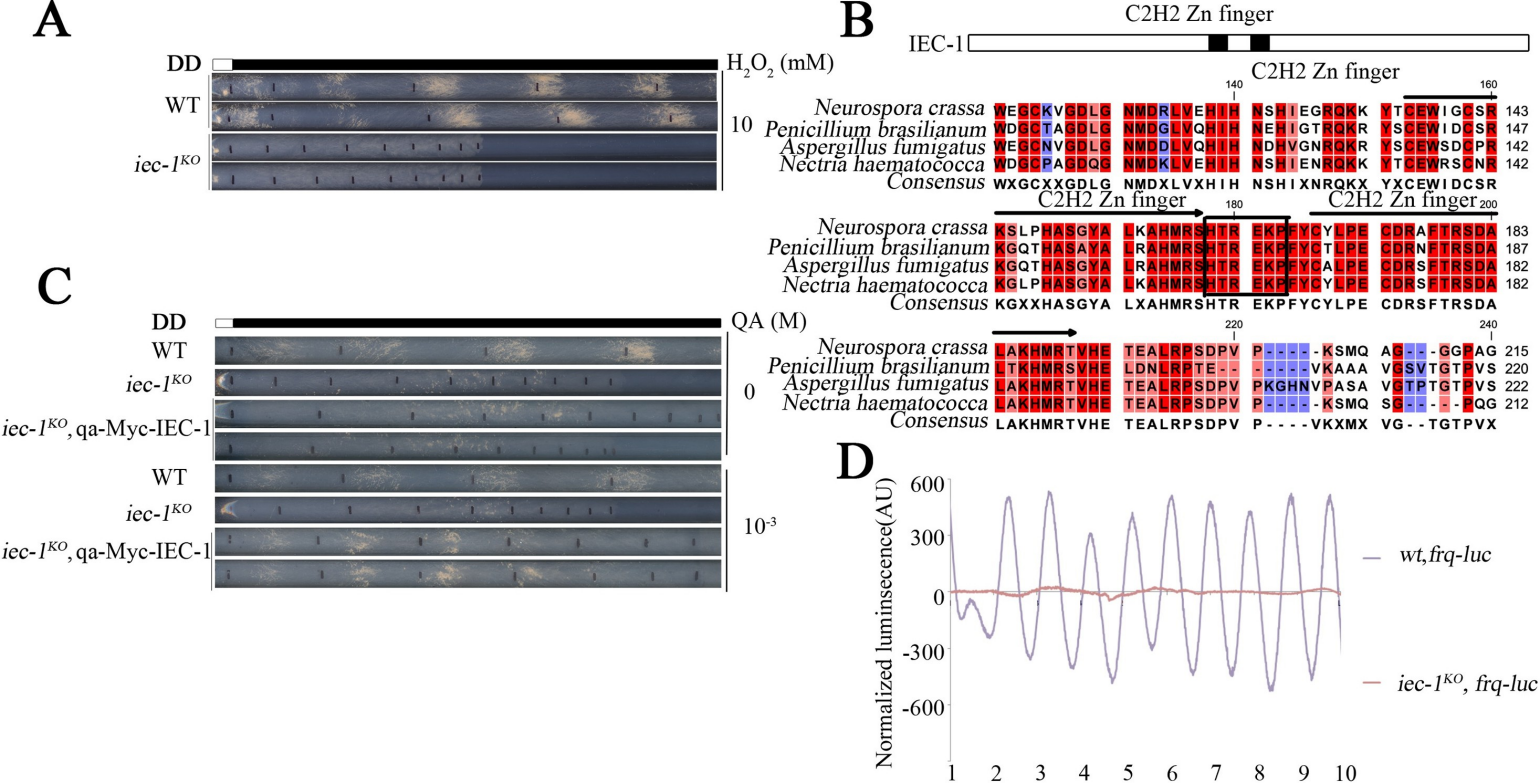
IEC-1 is required for normal circadian clock function. (A) Race tube assays of the wild-type and *iec-1*^*KO*^ strains. (B) Amino acid sequence alignment of the zf-C2H2 domains of IEC-1 from *Neurospora crassa*, *Penicillium brasilianum*, *Aspergillus fumigates* and *Nectria haematococca*. (C) Race tube assays of the wild-type strain, *iec-1*^*KO*^ strain, and *iec-1*^*KO*^, qa-Myc-IEC-1 transformants in a race tube with or without QA. Growth media on the race tubes did not consist of glucose. (D) Luciferase reporter assay showing the *frq* promoter activity in the *wt*, *frq-luc* and *iec-1*^*KO*^, *frq-luc* strains grown in DD for several days. Raw data were normalized to subtract the baseline calculated by the LumiCycle analysis software.

To characterize the transcription factors that are involved in the regulation of *frq* expression, we examined the conidiation rhythms of available knockout transcription factor mutants with race tube assays. As shown in Fig 1A, the strain with deletion of the *iec-1* (*ras-1*^+^) (NCU03206) gene showed arrhythmic conidiation in a race tube assay compared to the wild-type strain, suggesting that IEC-1 plays a critical role in *Neurospora* circadian conidiation rhythm. The *iec-1* gene codes a C2H2 finger domain-containing protein IEC-1, a homolog of mammalian Yin Yang 1 (YY1), *Drosophila* PcG PHO, and yeast Iec1 [39]. When the IEC-1 protein sequence was used in a BLAST search against protein databases, its homologs were found to be highly conserved in ascomycetes (Fig 1B). To confirm the phenotype of the *iec-1* mutant, the *iec-1* gene, which is driven by the quinic acid (QA)-inducible *qa*-2 promoter, was reintroduced to the *iec-1*^*KO*^ strain. In addition, five copies of the c-Myc epitope with six histidine residues [42] were inserted at the N-terminus of the IEC-1 ORF to facilitate the detection of the expression of Myc-IEC-1 using a c-Myc monoclonal antibody (9E10). In QA-containing race tubes, the circadian conidiation rhythms of the *iec-1*^*KO*^, qa-Myc-IEC-1 strains were similar to those of the wild-type strains (Fig 1C). These data indicate that Myc-IEC-1 can partially complement the function of the endogenous IEC-1 protein in the *iec-1*^*KO*^ strain. To test whether light exposure could entrain the conidiation rhythms, the wild-type strain and *iec-1*^*KO*^ strains were assayed under light/dark cycles (LD) at 25°C. The race tube assays showed that the conidiation process of the *iec-1*^*KO*^ strains was still entrained by LD cycles (S1A Fig). Meanwhile, a reduced light induction of FRQ expression by light was observed in the *iec-1* mutant (S1B Fig). Notably, the basal level of FRQ is higher in the mutant. Nonetheless, this result suggests that IEC-1 also affect the light induction of FRQ expression. These data suggested that the clock phenotype of the mutant strain is not caused by a defect in the input pathway of clock. To further confirm the arrhythmic phenotype of the *iec-1*^*KO*^ strain, we introduced bioluminescence reporter constructs (*frq*-*luc*) into the *his-3* locus of the *iec-1*^*KO*^ strains and the wild-type strains, in which the luciferase expression of both strains are driven by the *frq* promoter [43]. The sequence analysis of genomic DNA showed that the *frq* promoters in the luciferase-expressing strains were intact (S2 Fig). Consistent with the phenotype in the race tube assay, the robust bioluminescence rhythm of the *wt*, *frq-luc* strain was abolished in the *iec-1*^*KO*^, *frq-luc* strains (Fig 1D and S3A Fig), indicating that IEC-1 is critical for circadian clock function at the molecular level.

### IEC-1 suppresses *frq* transcription and rhythmically associates with the *frq* promoter

**Fig 2:**
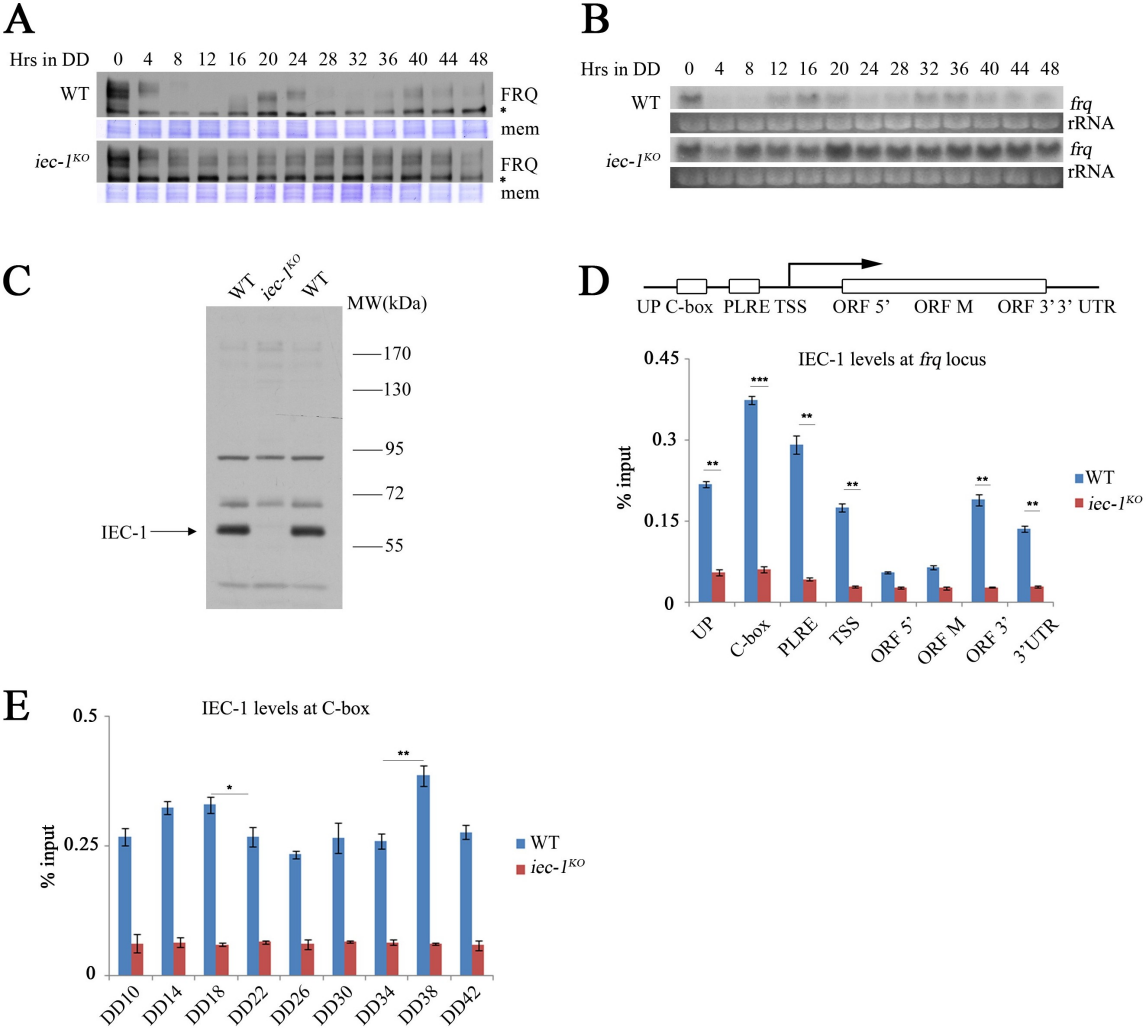
IEC-1 suppresses *frq* transcription and rhythmically binds to the *frq* promoter. (A) Western blot analysis showing the circadian oscillation of FRQ proteins in the wild-type and *iec-1*^*KO*^ strains. The strains were grown in 2% glucose liquid media. The asterisk indicates a nonspecific cross-reacted protein band recognized by our FRQ antiserum. The Coomassie Brilliant Blue-stained membranes (mem) represent the total protein in each sample and were used as a loading control. (B) Northern blot analysis of *frq* transcription in the wild-type and *iec-1*^*KO*^ strains. rRNA was used as a loading control. The strains were grown in 2% glucose liquid media. (C) Immunodetection of IEC-1 protein in the wild-type strain and the *iec-1*^*KO*^ mutant using antiserum that specifically recognizes the IEC-1 protein in the wild-type strain. The arrow notes the specific IEC-1 protein band detected by our IEC-1 antibody. The strains were grown in 2% glucose liquid media. (D) ChIP analysis showing the recruitment of IEC-1 at different regions of the *frq* locus in the wild-type and *iec-1*^*KO*^ strains at DD18. The strains were grown in 2% glucose liquid media. C-box, clock box; PLRE, proximal light-regulated element; TSS, transcription start site; ORF, open reading frame; UTR, untranslated region. (E) ChIP analysis showing the enrichment of IEC-1 at the C-box of the *frq* promoter in the wild-type and *iec-1*^*KO*^ strains at the indicated time points. The strains were grown in 2% glucose liquid media. Significance was assessed by two-tailed t-test. *P<0.05, **P<0.01. Error bars show the mean ±S.D. (n = 3).

To further determine the role of IEC-1 in the circadian clock, we examined the FRQ expression profile at different time points in constant darkness (DD). Both FRQ rhythms and FRQ phosphorylation profiles were disrupted in the *iec-1*^*KO*^ strain (Fig 2A). Northern blot analyses showed that the abolished FRQ rhythms were due to constant transcription of the *frq* gene in the *iec-1*^*KO*^ strain (Fig 2B). To test whether IEC-1 directly regulates *frq* transcription, a chromatin immunoprecipitation (ChIP) assay was performed by using an IEC-1-specific polyclonal antibody (Fig 2C). Because the IEC-1 antibody recognizes nonspecific bands, the *iec-1*^*KO*^ strain was used as the negative control in the ChIP assays. The ChIP assay revealed enrichment of IEC-1 at the *frq* promoter, ORF 3’, and the 3’UTR but not at ORF 5’ and the middle regions at DD18 (Fig 2D). Furthermore, the association of IEC-1 with C-box fluctuated from DD10 to DD42 (Fig 2E). Altogether, these results indicate that IEC-1 functions at the *frq* locus to regulate *frq* transcription.

### The INO80 complex is required for normal circadian conidiation rhythm and rhythmic *frq* transcription

Previous studies showed that yeast Iec1or mammalian Yin Yang 1 (YY1) co-purified with the INO80 complex in yeast or mammalian cells [39,44]. These transcription factors are required for the recruitment of the INO80 complex to target genes. Since the INO80 complex acts as a conserved co-factor of Iec1/YY1 in different organisms, we performed an IP assay to test the association of the INO80 complex with IEC-1 in *Neurospora*. The results showed that FLAG-IEC-1 was immunoprecipitated (IP) with INO80 (NCU08919) by the INO80 antiserum but not immunoprecipitated with the preimmune (PI) serum (S4A Fig). In the INO80 complex, INO80 is the catalytic subunit and IES-1 is a structural subunit [33,37,45]. To further identify whether the INO80 complex functions in the same pathway as IEC-1 in the regulation of the *Neurospora* circadian clock, we generated *ino80* (NCU08919) and *ies-1* (NCU01362) deletion mutants. As expected, both *ino80*^*KO*^ and *ies-1*^*KO*^ strains exhibited arrhythmic conidiation phenotypes in the race tube assays (S4B Fig). We also tried to generate mutants with a *band* background (*ras-1*^*bd*^), but we only obtained heterokaryotic strains. These strains also showed arrhythmic conidiation phenotypes in the race tube assays (S4 Fig). To further confirm that deletion of *ino80* or *ies-1* is the only cause of the clock defect in these mutants, rescue strains of the mutants were generated. The conidiation rhythms of the *ino80*^*KO*^, qa-Myc-INO80 and *ies-1*^*KO*^, qa-Myc-IES-1 transformants were not restored in the race tubes in the absence of QA (S4C and S4D Fig). Meanwhile, obvious conidiation rhythms were observed in the rescue strains but not in the knock-out strains in the race tube assays with 10^−3^ M QA (S4C and S4D Fig). However, we also observed a phase difference between the wild-type and the rescue strains. The results suggest that *qa-2* promoter-driven expression of Myc-INO80 could not fully complement the function of endogenous INO80 due to an abnormal expression level or the presence of the Myc tag. The race tube assays showed that the conidiation processes of the *ino80*^*KO*^ and *ies-1*^*KO*^ strains are entrained by LD cycles, excluding the defect in the input pathway of clock in these mutants (S4E Fig). Notably, the growth rates of *ino80*^*KO*^ strains were strongly dependent on glucose, indicating that *ino80*^*KO*^ strain might be sensitive to glucose or carbon starvation. Bioluminescence rhythms in *ino80*^*KO*^, *frq-luc* and *ies-1*^*KO*^, *frq-luc* strains were also abolished (S4F, S3B and S3C Figs). Western blots showed disruption of both the FRQ rhythms and FRQ phosphorylation profiles in the *ino80*^*KO*^ and *ies-1*^*KO*^ strains (S4G Fig). Northern blot analyses showed that the abolished FRQ rhythms were due to constant transcription of the *frq* gene in the *ino80*^*KO*^ and *ies-1*^*KO*^ strains (S4H Fig). Altogether, these results indicate that the INO80 complex is critical for *frq* transcription repression.

### IEC-1- and WCC-driven *frq* transcription are responsible for the recruitment of INO80 at the *frq* C-box

**Fig 3:**
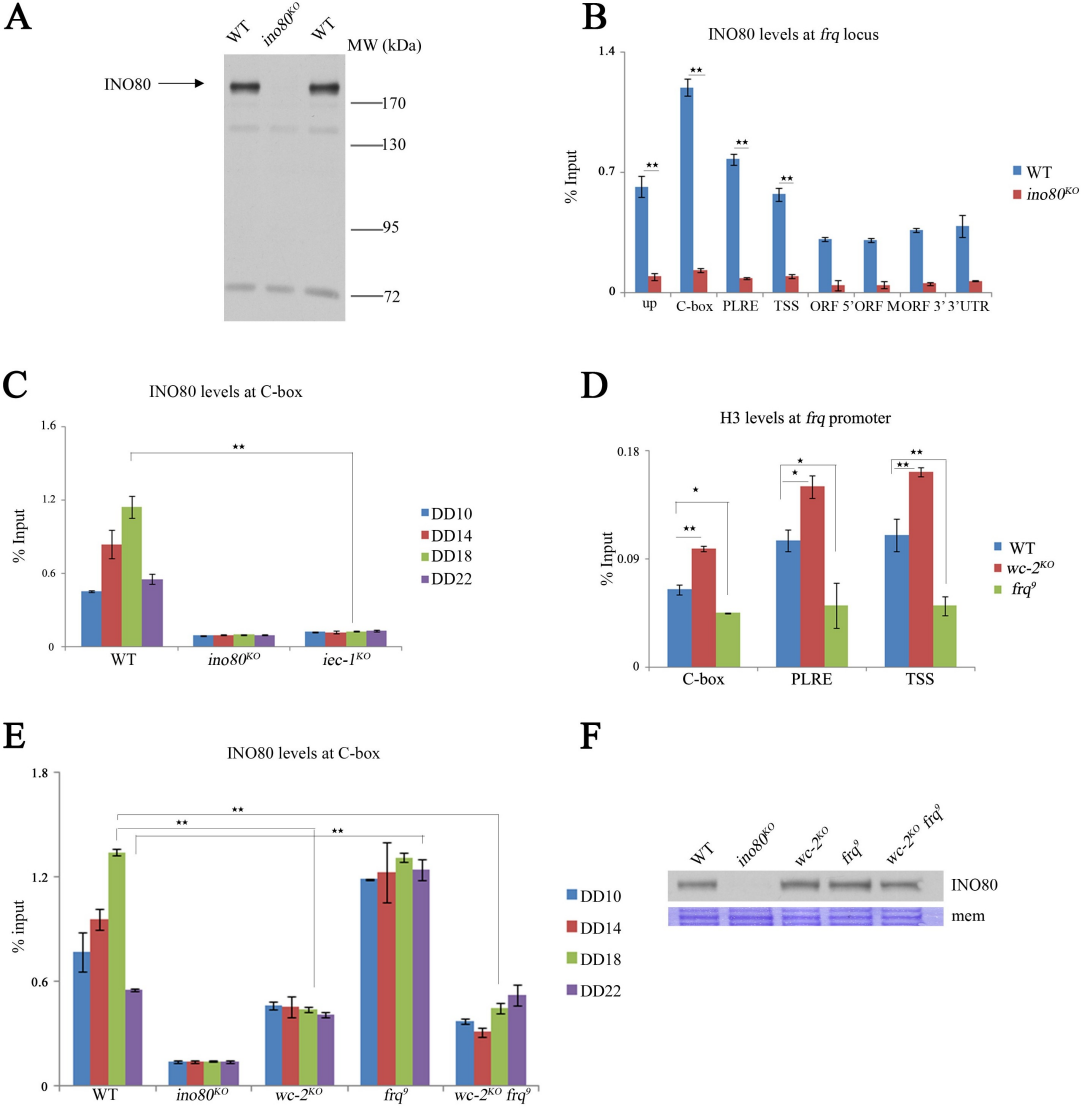
INO80 is rhythmically recruited at the C-box by IEC-1 and WCC-driven *frq* expression. (A) Immunodetection of INO80 in the wild-type strain and the *ino80*^*KO*^ mutant using antiserum that specifically recognizes INO80 protein in the wild-type strain. The strains were grown in 2% glucose liquid media. The arrow indicates the INO80 specific band in the wild-type strains. (B) ChIP analysis showing the recruitment of INO80 at different regions of the *frq* locus in the wild-type and *ino80*^*KO*^ strains at DD18. The *ino80*^*KO*^ strain was used as a negative control in the ChIP assay. The strains were grown in 2% glucose liquid media. Significance was assessed by two-tailed t-test. *P<0.05, **P<0.01. Error bars show the mean ±S.D. (n = 3). (C) ChIP analysis showing the recruitment of INO80 at the C-box in the wild-type, *ino80*^*KO*^ and *iec-1*^*KO*^ strains at the indicated time points. The strains were grown in 2% glucose liquid media. Significance was assessed by two-tailed t-test. *P<0.05, **P<0.01. Error bars show the mean ±S.D. (n = 3). (D) ChIP analysis showing the enrichment of histone H3 at the C-box, PLRE or TSS of the *frq* promoter region in the wild-type, *wc-2*^*KO*^ (*bd*) and *frq*^*9*^ (*bd*) mutant strains. The strains were grown in 2% glucose liquid media. Significance was assessed by two-tailed t-test. *P<0.05, **P<0.01. Error bars show the mean ±S.D. (n = 3). (E) ChIP analysis showing the recruitment of INO80 at the C-box of the *frq* promoter in the wild-type, *ino80*^*KO*^, *wc-2*^*KO*^(*bd*), *frq*^*9*^(*bd*), and *wc-2*^*KO*^ *frq*^*9*^ (*bd*) strains at the indicated time points. The strains were grown in 2% glucose liquid media. Significance was assessed by two-tailed t-test. *P<0.05, **P<0.01. Error bars show the mean ±S.D. (n = 3). (F) Western blot analysis showing the INO80 protein levels in the wild-type, *ino80*^*KO*^, *wc-2*^*K*O^ (*bd*), *frq*^*9*^ (*bd*) and *wc-2*^*KO*^ *frq*^*9*^ (*bd*) strains. The strains were grown in 2% glucose liquid media.

Previous studies have shown that the INO80 complex can be recruited to target genes by Iec1 or YY1 in yeast or mammalian cells [39,40]. To investigate the occupancy of the INO80 complex at the C-box of the *frq* gene, we performed a ChIP assay using INO80-specific antiserum at DD18 (Fig 3A). The ChIP results revealed that binding of INO80 to the *frq* gene peaks at the C-box and 3’-UTR (Fig 3B), similar to the binding pattern of IEC-1 (Fig 2D). Next, we assessed whether IEC-1 is required for recruitment of the INO80 complex at the C-box of the *frq* promoter. The ChIP assays conducted with an INO80 antibody showed a dramatic decrease in enrichment of INO80 at the C-box in *iec-1*^*KO*^ strains (Fig 3C), indicating that efficient binding of the INO80 complex at the *frq* C-box is dependent on the expression of IEC-1.

To determine whether the association of INO80 with the C-box is rhythmic during circadian cycles, the recruitment of INO80 at four different time points in constant darkness were examined using a ChIP assay. The results demonstrated that the association of INO80 with the C-box of the *frq* promoter was rhythmic, peaking at DD18 (Fig 3C). In yeast or mammalian systems, recruitment of the INO80 complex by Iec1 or YY1 is a key event in gene expression [39,40]. Thus, these results suggest that IEC-1-dependent INO80 recruitment at the *frq* promoter is required for suppression of *frq* transcription after WCC-inactivation by FRQ. In the *Neurospora* circadian system, the transcriptional activators WC-1 and WC-2 are responsible for the transcriptional activation of the *frq* gene during circadian cycles [7,9–11,13,46,47]. In the *frq*^*9*^ mutant, constant *frq* transcription [48] causes a low H3 density at the *frq* promoter, whereas, in the *wc-2*^*KO*^ strain, a lack of *frq* transcription [10] is associated with a high H3 density at the *frq* promoter (Fig 3D). These findings indicate that WC-mediated transcriptional activation leads to open chromatin states by nucleosomal removal, which increases the accessibility of the *frq* promoter. It is also possible that WC-mediated transcriptional activation of *frq* is required for rhythmic recruitment of the INO80 complex during the period of high expression of *frq* to prepare for repression of *frq* transcription. To assess this possibility, we examined enrichment of the INO80 complex at the *frq* C-box in the *wc-2*^*KO*^ and *frq*^*9*^ strains. The ChIP results showed that the levels of INO80 enrichment in the *wc-2* mutant were lower than that in the wild-type strain at the DD18 time point (Fig 3E). These data suggest that the rhythmic recruitment of the INO80 complex is dependent on WC-mediated transcriptional activation of *frq*. In contrast, in *frq*^*9*^ strains, the constant elevation of the levels of INO80 enrichment are due to the constant high elevation of *frq* expression, which is caused by malfunction of the negative feedback loop (Fig 3E). To verify that the high levels of recruitment of the INO80 complex in the *frq*^*9*^ mutant are due to the activity of the WC complex, we generated the *wc-2*^*KO*^ *frq*^*9*^ double-mutant strain, and examined the recruitment of INO80 at the C-box. Loss of WC-2 restored the low levels of recruitment of INO80 at the C-box in the *frq*^*9*^ mutant (Fig 3E). Meanwhile, the INO80 expression of these mutants was similar to that of the wild-type strain (Fig 3F). These data indicate that the highly activated transcription of *frq* by the WC complex also contributes to the rhythmic recruitment of the INO80 complex to the C-box of *frq* for subsequent suppression of *frq* transcription during circadian cycles.

### WC-independent *frq* transcription occurs in the *ies-1*^*KO*^ strain

**Fig 4:**
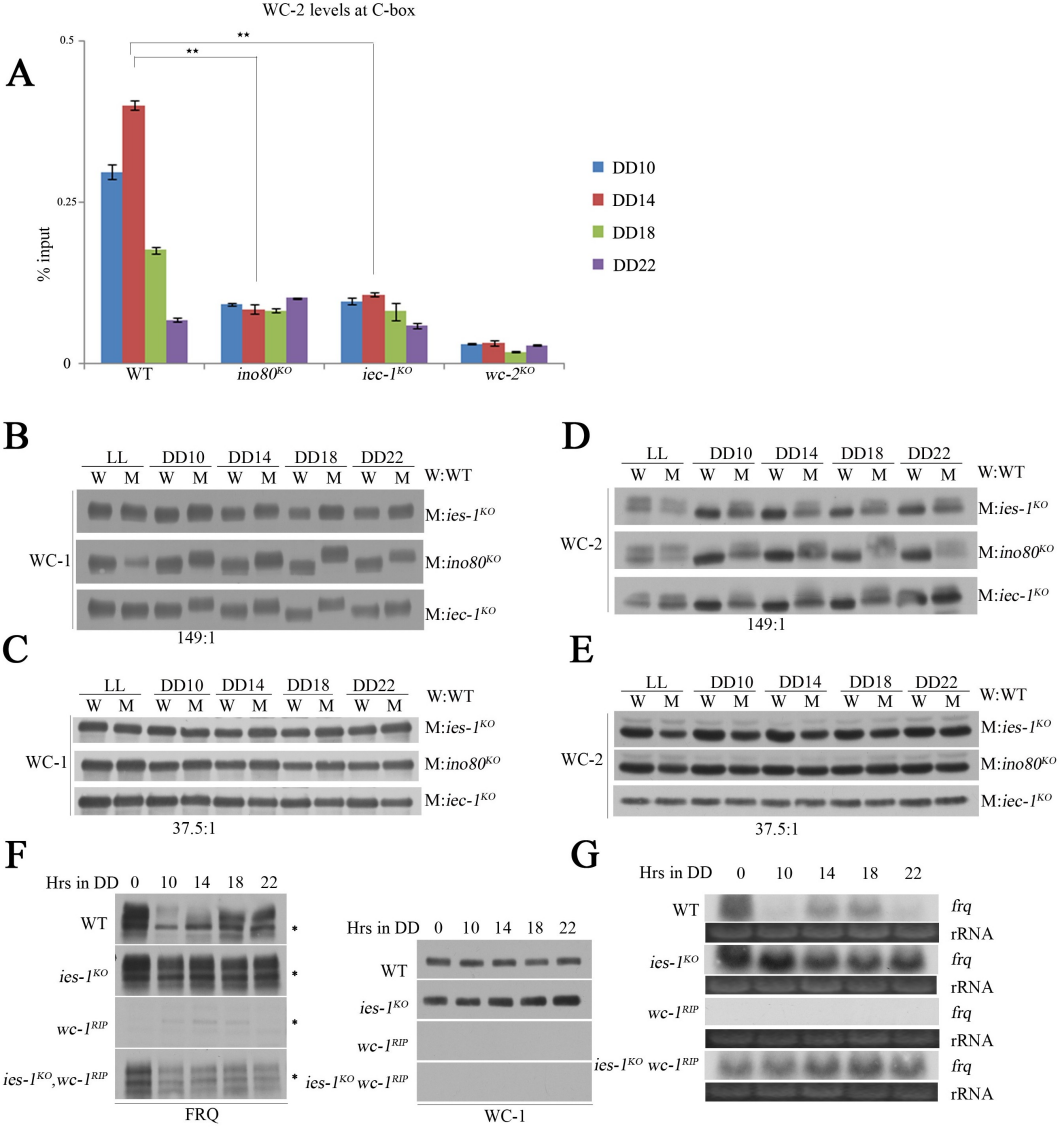
The INO80 complex is required for the suppression of WC-independent *frq* transcription. (A) ChIP analysis showing WC-2 enrichment at the C-box in the wild-type, *ino80*^*KO*^, *iec-1*^*KO*^, and *wc-2*^*KO*^ (*bd*) strains. The strains were grown in 2% glucose liquid media. Significance was assessed by two-tailed t-test. *P<0.05, **P<0.01. Error bars show the mean ±S.D. (n = 3). (B) Western blot analysis showing the phosphorylation of WC-1 in the wild-type, *ies-1*^*KO*^, *ino80*^*KO*^ and *iec-1*^*KO*^ strains. The numbers indicate the ratio of acrylamide/bisacrylamide used in the SDS-PAGE gel. The strains were grown in 2% glucose liquid media. (C) Western blot analysis showing the levels of WC-1 in the wild-type, *ies-1*^*KO*^, *ino80*^*KO*^ and *ies-1*^*KO*^ strains. The strains were grown in 2% glucose liquid media. (D) Western blot analysis showing the phosphorylation of WC-2 in the wild-type, *ies-1*^*KO*^, *ino80*^*KO*^ and *ies-1*^*KO*^ strains. The strains were grown in 2% glucose liquid media. (E) Western blot analysis showing the levels of WC-2 in the wild-type, *ies-1*^*KO*^, *ino80*^*KO*^ and *ies-1*^*KO*^ strains. The strains were grown in 2% glucose liquid media. (F) Western blot analysis of FRQ or WC-1 in the wild-type, *ies-1*^*KO*^, *wc-1*^*RIP*^ (*bd*), and *ies-1*^*KO*^ *wc-1*^*RIP*^ strains. The strains were grown in 2% glucose liquid media. (G) Northern blot analysis showing the levels of *frq* mRNA in the wild-type, *ies-1*^*KO*^, *wc-1*^*RIP*^ (*bd*), and *ies-1*^*KO*^ *wc-1*^*RIP*^ strains. The strains were grown in 2% glucose liquid media.

In the *Neurospora* circadian system, the WC complex, the positive transcription factor that binds to the C-box of *frq*, is responsible for rhythmic *frq* expression [7–9,13]. To test whether *frq* transcription is driven by the WC complex in *ino80* and *iec-1* mutants, we first examined the transcriptional activity of WC-2 in the *ino80*^*KO*^ and *iec-1*^*KO*^ strains. The ChIP results revealed that the rhythmic enrichment of WC-2 at the *frq* C-box corresponded to the rhythmic expression of *frq* in the wild type strain (Fig 4A). In contrast, the recruitment of WC-2 was dramatically decreased in the *ino80*^*KO*^ and *iec-1*^*KO*^ strains (Fig 4A), which was inconsistent with the constant expression of *frq* in these mutants. This finding suggests that the transcriptional activities of WC-1 and WC-2 are decreased in the *ino80* and *iec-1* mutants. Previous studies showed that hypophosphorylated WC-1 and WC-2 efficiently bind to the C-box for *frq* transcriptional activation [12,21] while hyperphosphorylated WC-1 and WC-2 exhibit lower binding activity at the C-box of *frq* [14,49]. Western blot analysis showed that the phosphorylation levels of WC-1 and WC-2 were increased in the *ino80*^*KO*^, *ies-1*^*KO*^ and *iec-1*^*KO*^ strains compared to those in the wild-type strains at different time points in DD (Fig 4B and 4D). In addition, we found no significant changes in the protein levels of WC-1 and a slight decrease in the levels of WC-2 in the *ino80*^*KO*^, *ies-1*^*KO*^ and *iec-1*^*KO*^ strains compared to those in the wild-type strains (Fig 4C and 4E). The levels of WC-1 protein did not exhibit a robust circadian rhythm in our hands, which is consistent with previous studies showing that the amplitudes of WC rhythms are variable. Since WC activity is mostly known to be regulated by phosphorylation, the variability of the WC-1 rhythms might be due to the use of different WC-1 antibodies in different laboratories which may have different sensitivity to different isoforms of WC-1. These results indicate the increased *frq* transcripts in these mutants are not caused by the increase of transcriptional activity of WCC. These data also suggest that the high levels of *frq* transcription could be partially independent of WC expression in these mutants. To further confirm this possibility, we generated an *ies-1*^*KO*^ *wc-1*^*RIP*^ double mutant and compared its FRQ levels to that of a *wc-1* single mutant. Consistent with previous results, the FRQ levels were extremely low in the *wc-1* single mutant (Fig 4F) [10]. However, the levels of FRQ protein and *frq* mRNA in the *ies-1*^*K*O^ *wc-1*^*RIP*^ double mutant were well detected with Western blot or Northern blot analyses (Fig 4F and 4G), indicating that WC-independent *frq* transcription exists in the *ies-1* mutants. Altogether, these results demonstrate that recruitment of the INO80 complex at the C-box through high transcriptional activation prepares for suppression of *frq* transcription after WCC inactivation.

### The INO80 complex contributed to the establishment of the dense chromatin environment at the *frq* promoter

**Fig 5:**
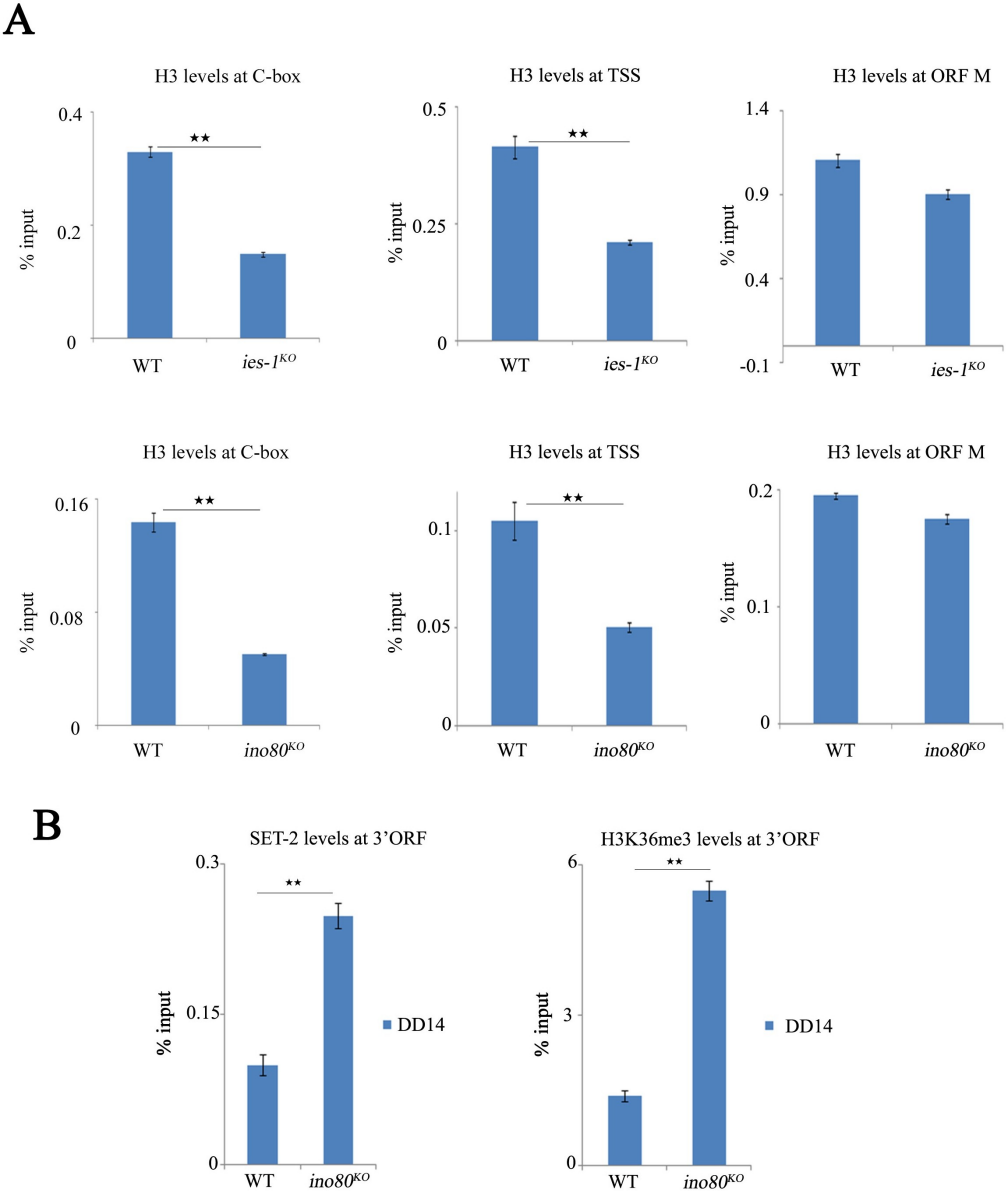
The establishment of nucleosomal barriers at the *frq* promoter by the INO80 complex prevents RNA pol II initiation. (A) ChIP analysis showing H3 density at the C-box, TSS or ORF middle regions of the *frq* locus in the wild-type, *ies-1*^*KO*^, and *ino80*^*KO*^ strains. The strains were grown in 2% glucose liquid media. Significance was assessed by two-tailed t-test. *P<0.05, **P<0.01. Error bars show the mean ±S.D. (n = 3). (B) ChIP analysis showing the recruitment of SET-2 and enrichment of H3K36me3 at the ORF 3’ of *frq* in the wild-type and *ino80*^*KO*^ strains. The strains were grown in 2% glucose liquid media. Significance was assessed by two-tailed t-test. *P<0.05, **P<0.01. Error bars show the mean ±S.D. (n = 3).

In *S*. *cerevisiae*, the first four nucleosomes at the transcription start site of genes have critical roles in the process of transcription initiation [50]. In *Drosophila*, the +1 nucleosome strongly inhibits the normal function of RNA polymerase II [51]. Although the INO80 complex occupied and peaked at the boundary of the genes, genome-wide ultra-high-resolution ChIP-exo data showed that the Arp5 subunit of the INO80 complex was particularly enriched at the +1 position in yeast [36,52]. Antisense transcripts *qrf* in the *Neurospora* circadian system revealed that induction of *qrf* promotes *frq* gene expression by creating a more accessible local chromatin environment, even in the absence of the WC complex [28]. Given that *qrf*-induced *frq* transcripts share the same properties with the WC-independent *frq* transcripts, the chromatin states in these two pathways should be similar. The ChIP assay showed that the H3 levels were dramatically reduced at both the C-box and TSS in the *ino80*^*KO*^ and *ies-1*^*KO*^ strains compared to that of the wild-type strains (Fig 5A). However, the H3 levels at the *frq* ORF in mutants were similar to that of the wild-type strains (Fig 5A). These results indicate that nucleosome density is decreased at the C-box and TSS of the *frq* gene in mutants.

SET-2-mediated H3K36me3 is an important landmark on chromatin during transcription elongation [53]. To confirm the enhanced transcription of *frq* induced by the decreased nucleosomal barrier in the *ino80*^*KO*^ strains, a ChIP assay was performed to examine the recruitment of SET-2 and enrichment of H3K36me3 at the 3’ORF of the *frq* gene. As expected, the recruitment of SET-2 and the enrichment of H3K36me3 were significantly increased at the 3’ ORF of the *frq* gene in the *ino80*^*KO*^ strains compared to those of the wild-type strains (Fig 5B). Together, these results demonstrate that the INO80 complex contributes to establishment of the dense chromatin environment at the *frq* promoter, which is essential for suppressing WC-independent *frq* transcription.

## Discussion

In this study, we identified a C2H2 finger domain-containing protein IEC-1 and its co-factor, the INO80 complex, which as part of their normal cellular roles in chromatin assembly, facilitate generalized repression of the clock gene *frq* in *Neurospora*. To investigate the role of IEC-1 and the INO80 complex, we generated the *iec-1*^*KO*^, *ino80*^*KO*^ and *ies-1*^*KO*^ strains and discovered that IEC-1 and the INO80 complex are required for normal circadian clock function. Based on the data presented in this manuscript, the INO80 complex is rhythmically recruited by IEC-1 to the *frq* promoter to suppress *frq* expression in the *Neurospora* clock. The recruitment of the INO80 complex to the *frq* locus by the transcription factor, IEC-1, is important for *frq* transcriptional repression, since the disruption of INO80 binding to the *frq* promoter in the *iec-1*^*KO*^, *ino80*^*KO*^ and *ies-1*^*KO*^ strains leads to high FRQ levels and loss of *frq* rhythmicity (Fig 2 and S4 Fig). A similar situation was observed in deletion of the transcriptional co-repressor RCO-1-RCM-1 [54,55]. Rhythmic activation and repression of *frq* transcription are required for the function of the *Neurospora* circadian clock. Therefore, normal suppression of *frq* expression is essential for the circadian auto-regulatory feedback loop function in the *Neurospora* clock. Nucleosomes at the TSS were identified as the strongest barriers for RNA Polymerase II in *S*. *cerevisiae* and *Drosophila* [50,51]. The FRQ expression levels in the *iec-1*^*KO*^, *ino80*^*KO*^ and *ies-1*^*KO*^ strains were high and arrhythmic (Fig 2 and S4 Fig), suggesting that the recruitment of the INO80 complex is a key step for establishing repressed state of chromatin at the *frq* promoter. Our ChIP data revealed that deletion of INO80 or IES-1 results in decreased nucleosome density at the *frq* promoter. These results suggest that the INO80 complex is required for establishing compact chromatin environments at the *frq* promoter.

We tried several times but failed to detect tight interaction between the WCC and the IEC-1-INO80 complex. However, weak or transient interaction between them might still occur. Our results showed that the rhythmic binding of the INO80 complex is also dependent on *frq* transcription activation by the WC complex in the wild-type strain (Fig 3E). Due to the constant transcriptional activation of *frq*, a significant decrease in nucleosome density along with increased recruitment of the INO80 complex at the *frq* promoter was observed in the *frq*^*9*^ mutant strains (Fig 3D and 3E). In contrast, the nucleosome density at the *frq* promoter was increased dramatically in the *wc-2*^*KO*^ strains because of the inactivation of *frq* transcription and reduced INO80 binding (Fig 3D and 3E). These results indicate that binding of the WC complex to the *frq* promoter results in an open chromatin state in this region and recruitment of the INO80 complex by IEC-1. The progressive inactivation of the WC complex by accumulated FRQ stimulates the remodeling activity of the INO80 complex associated with the *frq* promoter, which increases the nucleosome density at the *frq* promoter (Fig 5A). In contrast, although high levels of INO80 recruitment at the *frq* promoter were observed in the *frq*^*9*^ mutant, the INO80 complex could not re-assemble the dense chromatin state at the *frq* promoter in this mutant due to the absence of the FRQ protein, which is needed to shut down the high levels of *frq* transcription driven by the WC complex. In the wild type strain, the lowest level of INO80 recruitment was found at DD22. At this time point the *frq* transcripts decline and the *frq* promoter is condensed which is in a line with the data above. These data suggest that at DD22, the chromatin structure mediated by IEC-1 and INO80 complex to suppress *frq* transcription has already been established which is unsuitable for the binding of INO80 complex. Current results indicate that the FRQ-FRH complex functions as the negative element in the *Neurospora* circadian auto-regulatory feedback loop to inactivate the WC complex [12,14–16] and activate INO80 to achieve complete repression of *frq* expression. Thus, the open chromatin state of WCC-driven *frq* transcription promotes the recruitment of the INO80 complex, which closes the negative feedback loop upon WCC inactivation by FRQ. Similar to the *Neurospora* clock, the rhythmic activation of the clock genes in *Drosophila* and mammals by a heterodimeric PAS domain containing the transcription factors, CLK:CYC or the CLOCK:BMAL1 complex is essential for circadian clock function. In the mammalian clock, the binding of CLOCK:BMAL1 to the E-box of clock genes promotes nucleosome eviction and incorporation of the histone variant H2A.Z [29], which suggests that activation of the clock genes by CLOCK:BMAL1 leads to changes in the chromatin structures. Whether the open chromatin states of animal clock genes activated by CLK:CYC or CLOCK:BMAL1 can promote the binding of a remodeler to promoters of clock genes, similar to our results in *Neurospora*, is not clear. Considering the conserved roles of IEC1/PHO/YY1 and the INO80 complex in different organisms, it is worth determining whether there are similar mechanisms in *Drosophila* and mammals. Altogether, these results suggest that a conserved repression mechanism involving chromatin regulation exists in eukaryotic circadian systems.

## Materials and methods

### Strains and culture conditions

The wild-type strain (4200) was used as a control. The *iec-1* or *ies-1* genes were deleted by the replacement of their ORFs with a hygromycin resistance gene (*hph*) on the *ku70*^*RIP*^ (*bd*, *a*) background strain. The *ku70*^*RIP*^ *iec-1*^*KO*^ and *ku70*^*RIP*^ *ies-1*^*KO*^ strains were crossed with the 774–10 (A, *his-3*^-^) strain to obtain homokaryotic *iec-1*^*KO*^ and *ies-1*^*KO*^ strains, respectively. The *ku70*::*bar ino80*^*KO*^ strain was generated by the replacement of its ORF with the hygromycin resistance gene (*hph*) on the *ku70*::*bar* background strain and microconidia purification. The *ku70*::*bar ras-1*^*bd*^ *ino80*^*KO*^ and *ras-1*^*bd*^ *ino80*^*KO*^ strains were generated in the same manner as the *ku70*::*bar ino80*^*KO*^ strain. For rescue strains, the plasmid qa-5Myc-6his-IEC-1 was transformed into the *iec-1*^*KO*^ strain. The *ino80*^*KO*^, qa-5Myc-6his-INO80 and *ies-1*^*KO*^, qa-5Myc-6his-IES-1 transformants were obtained in the same manner as the *iec-1*^*KO*^, qa-5Myc-6his-IEC-1 transformants. A plasmid containing the full-length *frq* promoter fused to luciferase was transformed into the *iec-1*^*KO*^, *ino80*^*KO*^ and *ies-1*^*KO*^ strains to generate the *iec-1*^*KO*^, *frq-luc*, *ino80*^*KO*^, *frq-luc* and *ies-1*^*KO*^, *frq-luc* strains. Liquid culture conditions were the same as a previously published method [56]. The *wc-2*^*KO*^, *frq*^*9*^, *wc-2*^*KO*^ *frq*^*9*^, *wc-1*^*RIP*^ and *ies-1*^*KO*^ *wc-1*^*RIP*^ all contain the band mutation.

### Race tube assay

The race tube medium contained 1× Vogel’s salts, 0.1% glucose, 0.17% arginine, 50 ng/mL biotin and 1.5% agar supplemented with or without 10 mM H_2_O_2_. Conidia of different strains were inoculated at one end of each race tube and were grown under constant light (LL) for 1 day to synchronize the clock. The race tubes were then transferred to constant darkness (DD), and the position of the advancing mycelia front was marked at 24 h intervals on the tube. When growth was completed, tubes were scanned, and the growth period of each strain was calculated.

### Generation of antiserum against INO80 and IEC-1

GST-INO80 (containing INO80 amino acids 1–341) and GST-IEC-1 (containing IEC-1 amino acids 9–175) fusion proteins were expressed in BL21 cells by induction of IPTG. After purification, the recombinant proteins were used as antigens to immunize rabbits, which yielded rabbit polyclonal antiserums [57].

### ChIP analysis

ChIP assay was performed as previously described [55]. *Neurospora* tissues were fixed by shaking in 1% formaldehyde for 15 min at 25°C, and cross-linking reactions were stopped by adding glycine at a final concentration of 125 mM. The cross-linked chromatin was sheared by sonication to approximately 200–500 bp fragments. A 1 mL aliquot of protein (2 mg/mL) was used per immunoprecipitation, and 10 μL was maintained as the input DNA. The chromatin immunoprecipitation reaction was carried out with 2 μL antibody to WC-2, 2.5 μL antibody to H3 (2650; Cell Signaling Technology), 2.5 μL antibody to IEC-1, 5 μL antibody to INO80, 5 μL antibody to SET-2, and 2 μL antibody to H3K36me3 (4909; Cell Signaling Technology). Immunoprecipitated DNA was quantified using real-time PCR. The primers for real-time PCR were designed according to a previously published protocol [27]. The ChIP-qPCR data were normalized by the input DNA, and the results were presented as the percentage of input DNA. Each experiment was independently performed at least three times.

### Protein analyses

Protein extraction, quantification and western blot analysis were performed as previously described [58,59]. Western blot analyses were performed by using antibodies against the proteins of interest. Equal amounts of total protein (40 μg) were loaded in each lane. After electrophoresis, proteins were transferred onto PVDF membranes, and western blot analysis was performed.

### RNA analyses

RNA was extracted by TRIzol [60] and analyzed by northern blotting as previously reported [56]. Shortly, equal amounts of total RNA (20 μg) were loaded onto agarose gels for electrophoresis, and the gels were blotted and probed with an RNA probe specific for *frq* mRNA.

### Luciferase reporter assay

The luciferase reporter assay was performed as previously reported [54]. The bioluminescence reporter construct (*frq-luc*), in which luciferase expression is driven by the *frq* promoter, was introduced into the *his-3* locus of the *iec-1*^*KO*^, *iec-1*^*KO*^, *ino80*^*KO*^ and wild-type strains. One drop of conidia suspensions in water was placed on AFV medium and grown in constant light (LL) overnight at 25°C. The cultures were then transferred to constant darkness, and luminescence was recorded in real time using LumiCycle after one day in DD at 25°C. The data were then normalized with LumiCycle analysis software by subtracting the baseline luciferase signal, which increases as the cells grow. Under our experimental condition, luciferase signals are highly variable during the first day in the LumiCycle but become stabilized afterwards, which is likely due to the light-dark transfer of the cultures. Thus, the results were recorded after one day in DD at 25°C.

## Supporting information

S1 Fig Examining of light induction of FRQ expression in *iec-1*^*KO*^ strain. (A) Race tube assays of the wild-type and *iec-1*^*KO*^ strains under light-dark cycles at 25°C. (B) Western blot analysis showing FRQ protein levels in the wild-type and *iec-1*^*KO*^ strains in the indicated time points after exposure to light from the samples in DD24.
(TIF)

S2 Fig Sequence analysis of the *frq* promoter in the luciferase-expressing strains. (TIF)

S3 Fig The absolute levels of luminescence. (A) Luciferase reporter assay showing the *frq* promoter activity in the *wt*, *frq-luc* and *iec-1*^*KO*^, *frq-luc* strains grown in DD for several days. (B) Luciferase reporter assay showing the *frq* promoter activity in the *wt*, *frq-luc* and *ies-1*^*KO*^, *frq-luc* strains grown in DD for several days. (C) Luciferase reporter assay showing the *frq* promoter activity in the *wt*, *frq-luc* and *ino80*^*KO*^, *frq-luc* strains grown in DD for several days.
(TIF)

S4 Fig The IEC-1-associated INO80 complex is a component of the *Neurospora* circadian clock. (A) IP analysis showing the interaction between INO80 and FLAG-IEC-1. The extracts of the *wt*, FLAG-IEC-1 strain were immunoprecipitated by the preimmune serum (PI) or the INO80 antiserum (IP), followed by western blot analysis using the FLAG or INO80 antibodies. The strains were grown in 2% glucose liquid media. (B) Race tube assays of the wild-type, *ino80*^*KO*^ and *ies-1*^*KO*^ strains. (C) Race tube assays of the wild-type, *ino80*^*KO*^, and *ino80*^*KO*^, qa-Myc-INO80 strains with or without QA. Growth media on the race tubes did not consist of glucose. (D) Race tube assays of the wild-type, *ies-1*^*KO*^, and *ies-1*^*KO*^, qa-Myc-IES-1 strains with or without QA. Growth media on the race tubes did not consist of glucose. (E) Race tube assays of the wild-type, *ino80*^*KO*^ and *iec-1*^*KO*^ strains under light-dark cycles at 25°C. (F) Luciferase reporter assays showing the *frq* promoter activity in the *wt*, *frq-luc*, *ino80*^*KO*^, *frq-luc* and *ies-1*^*KO*^, *frq-luc* strains grown in DD for several days. Raw data were normalized to subtract the baseline calculated by the LumiCycle analysis software. (G) Western blot analysis showing the circadian oscillation of FRQ in the wild-type, *ies-1*^*KO*^ and *ino80*^*KO*^ strains. The strains were grown in 2% glucose liquid media. The asterisk indicates a nonspecific cross-reacted protein band recognized by our FRQ antiserum. “mem” indicates the membrane stained by Coomassie Brilliant Blue used as a loading control. (H) Northern blot analysis showing the levels of *frq* mRNA in the wild-type, *ino80*^*KO*^ and *ies-1*^*KO*^ strains. rRNA was used as a loading control. The strains were grown in 2% glucose liquid media.
(TIF)

S5 Fig The circadian and molecular characterization of the heterokaryotic *ras-1*^*bd*^ *ino80*^*KO*^ (A), *ku70*::*bar ras-1*^*bd*^ *ino80*^*KO*^ (B), *ku70*^*RIP*^ *ras-1*^*bd*^ *ino80*^*KO*^ (C) and *ku70*::*bar ino80*^*KO*^ *(D)*. The *ino80*^*KO*^ strain was used as a positive control.
(TIF)
