# Supplementary figures and images for: Transcriptional repression of frequency by the IEC-1-INO80 complex is required for normal Neurospora circadian clock function
Source: PLoS Genet. 2017 Apr 12;13(4):e1006732. doi: 10.1371/journal.pgen.1006732 (PMC5406019; doi:10.1371/journal.pgen.1006732)

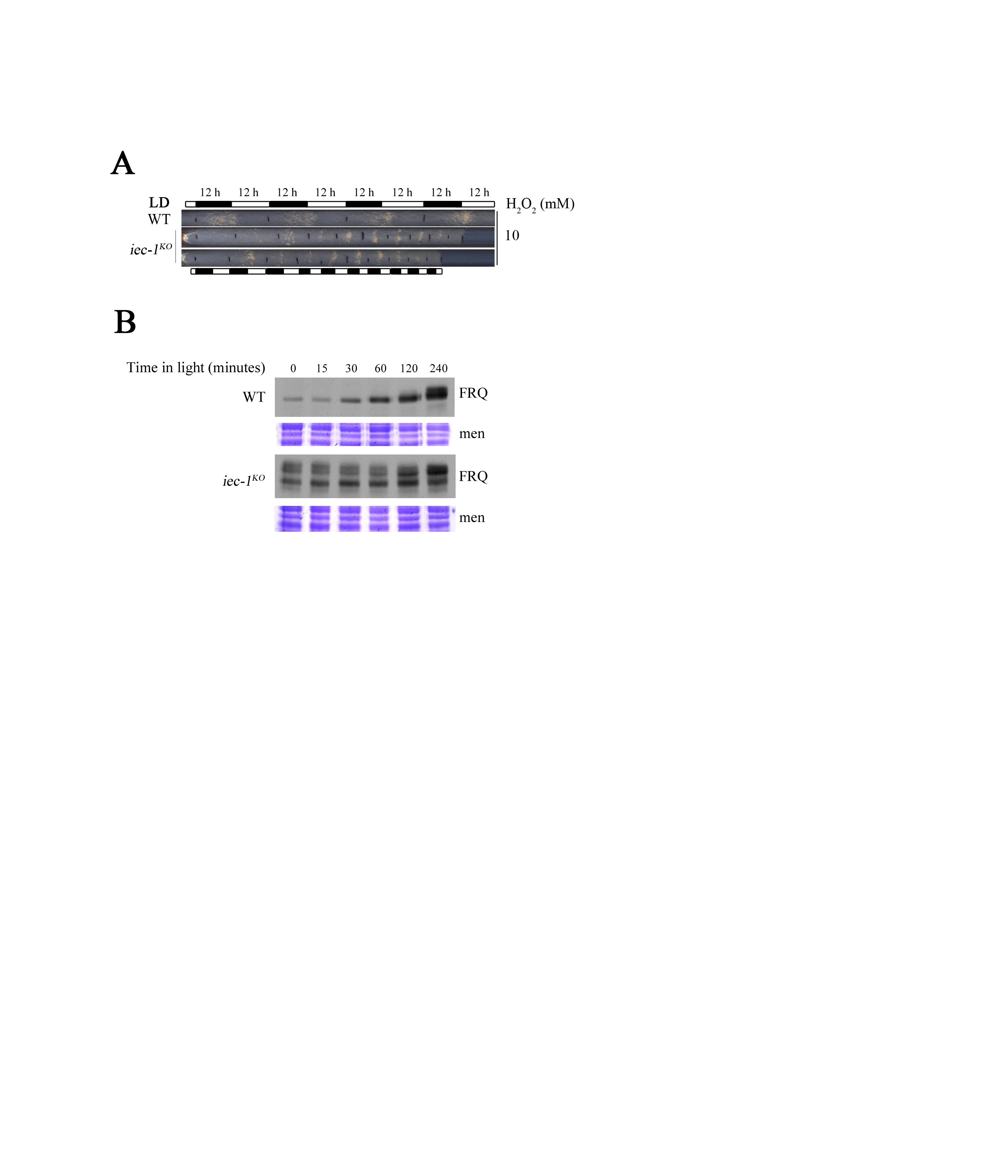

Supplement: S1 Fig — (A) Race tube assays of the wild-type and iec-1KO strains under light-dark cycles at 25°C. (B) Western blot analysis showing FRQ protein levels in the wild-type and iec-1KO strains in the indicated time points after exposure to light from the samples in DD24. (TIF) [file pgen.1006732.s001.tif]

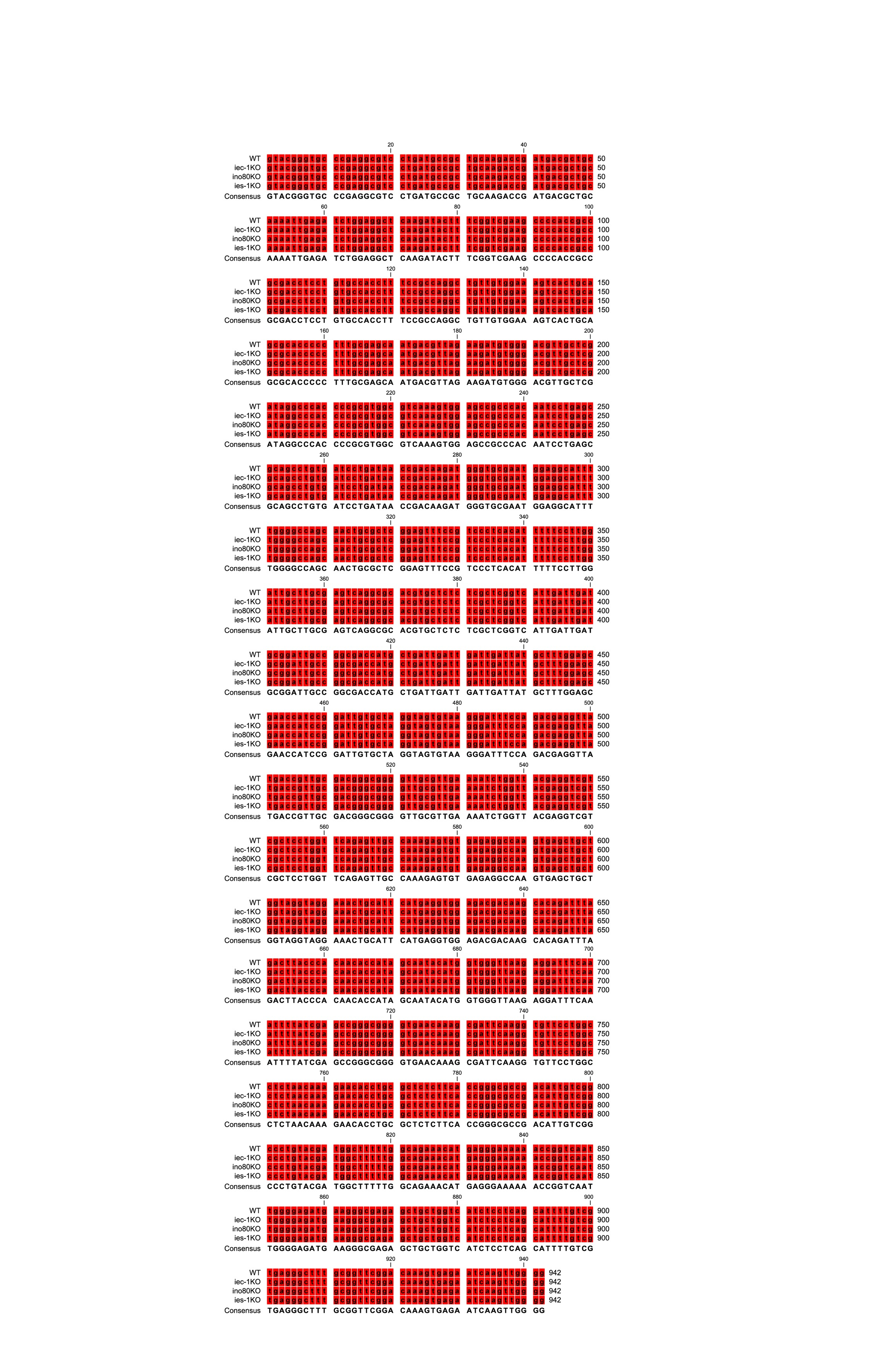

Supplement: S2 Fig — (TIF) [file pgen.1006732.s002.tif]

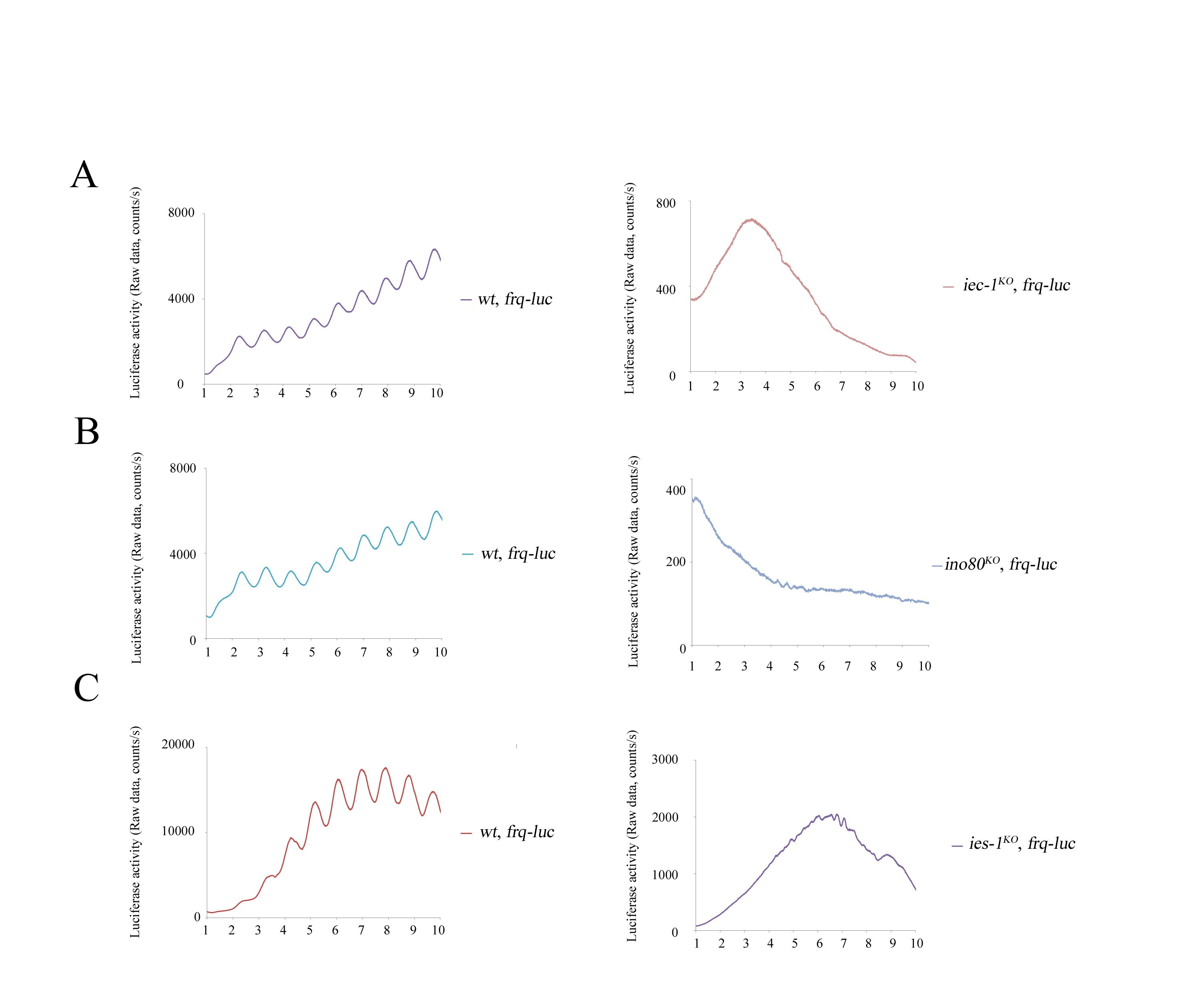

Supplement: S3 Fig — (A) Luciferase reporter assay showing the frq promoter activity in the wt, frq-luc and iec-1KO, frq-luc strains grown in DD for several days. (B) Luciferase reporter assay showing the frq promoter activity in the wt, frq-luc and ies-1KO, frq-luc strains grown in DD for several days. (C) Luciferase reporter assay showing the frq promoter activity in the wt, frq-luc and ino80KO, frq-luc strains grown in DD for several days. (TIF) [file pgen.1006732.s003.tif]

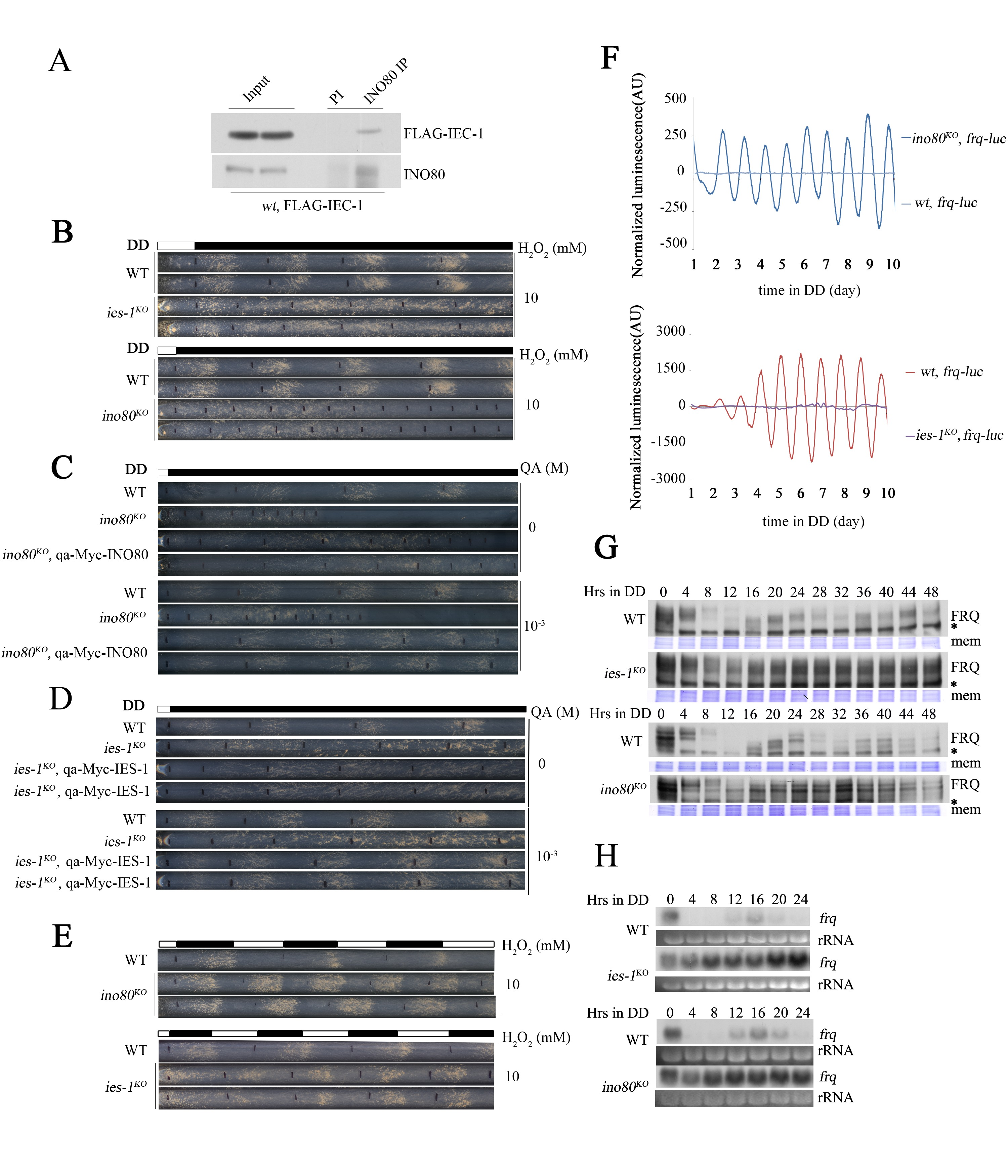

Supplement: S4 Fig — (A) IP analysis showing the interaction between INO80 and FLAG-IEC-1. The extracts of the wt, FLAG-IEC-1 strain were immunoprecipitated by the preimmune serum (PI) or the INO80 antiserum (IP), followed by western blot analysis using the FLAG or INO80 antibodies. The strains were grown in 2% glucose liquid media. (B) Race tube assays of the wild-type, ino80KO and ies-1KO strains. (C) Race tube assays of the wild-type, ino80KO, and ino80KO, qa-Myc-INO80 strains with or without QA. Growth media on the race tubes did not consist of glucose. (D) Race tube assays of the wild-type, ies-1KO, and ies-1KO, qa-Myc-IES-1 strains with or without QA. Growth media on the race tubes did not consist of glucose. (E) Race tube assays of the wild-type, ino80KO and iec-1KO strains under light-dark cycles at 25°C. (F) Luciferase reporter assays showing the frq promoter activity in the wt, frq-luc, ino80KO, frq-luc and ies-1KO, frq-luc strains grown in DD for several days. Raw data were normalized to subtract the baseline calculated by the LumiCycle analysis software. (G) Western blot analysis showing the circadian oscillation of FRQ in the wild-type, ies-1KO and ino80KO strains. The strains were grown in 2% glucose liquid media. The asterisk indicates a nonspecific cross-reacted protein band recognized by our FRQ antiserum. “mem” indicates the membrane stained by Coomassie Brilliant Blue used as a loading control. (H) Northern blot analysis showing the levels of frq mRNA in the wild-type, ino80KO and ies-1KO strains. rRNA was used as a loading control. The strains were grown in 2% glucose liquid media. (TIF) [file pgen.1006732.s004.tif]

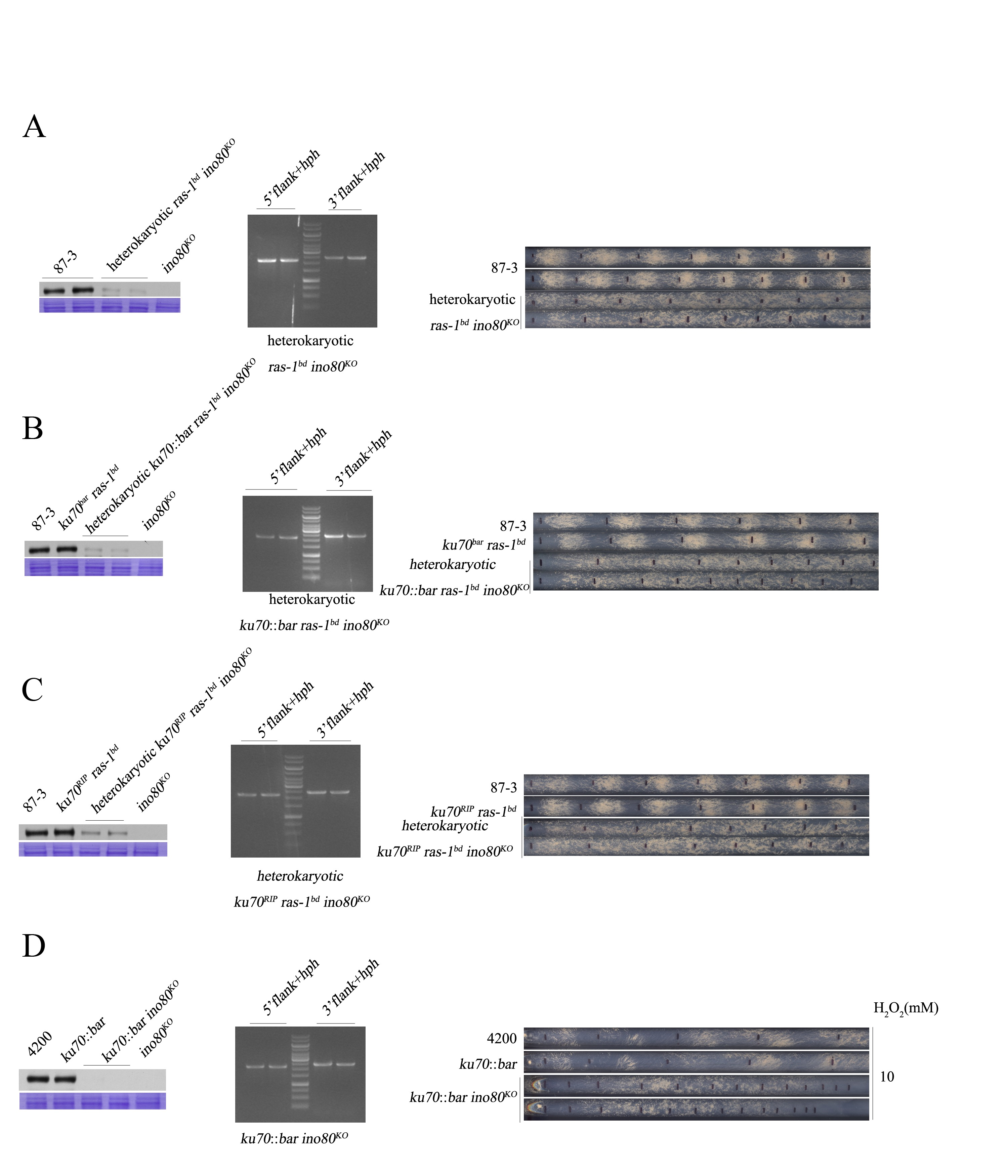

Supplement: S5 Fig — The ino80KO strain was used as a positive control. (TIF) [file pgen.1006732.s005.tif]
